# Shared Book Reading and Bilingual Children’s Dual Language Learning and Socio-Emotional Skills

**DOI:** 10.3390/bs16060886

**Published:** 2026-06-01

**Authors:** Qiujuan Cheng, He Sun

**Affiliations:** National Institute of Education, Nanyang Technological University, Singapore 637616, Singapore; nie24.cq@e.ntu.edu.sg

**Keywords:** shared book reading, home literacy environment, dual language, socio-emotional skills

## Abstract

In English-dominant Singapore, Chinese families make decisions about shared reading quantity, language allocation, and reading orientation at home. This study examined these three dimensions among 39 Singaporean Chinese families with children aged 5–6 years and their associations with children’s language abilities and socio-emotional outcomes. Families reported an English-dominant reading pattern (73.9% English vs. 26.1% Chinese). Although 92.3% of parents valued Chinese exposure, their ideal Chinese reading allocation (44.7%) substantially exceeded their actual practice (26.1%); parents also reported stronger behavioral than cognitive reading orientation. In exploratory regression analyses, Chinese weekly reading time and parental Chinese proficiency were positively associated with children’s Chinese language ability, although the overall Chinese model was marginal; the English language model did not reach significance, and English weekly reading time was not retained. For socio-emotional outcomes, reading orientation and children’s Chinese language ability were positively associated with prosocial behavior, whereas the disruptive behavior model did not provide evidence of a reliable association. These findings suggest that home shared reading may relate to bilingual children’s language and socio-emotional outcomes through input-related and interaction-related dimensions, but require cautious interpretation and replication in larger, longitudinal samples.

## 1. Introduction

In multilingual societies such as Singapore, young children grow up with unequal exposure to the languages in their environment. English functions as the principal medium of instruction and of public communication, while mother tongues such as Mandarin Chinese occupy a policy-endorsed role whose prominence varies considerably across households (Curdt-Christiansen, 2016; Li & Tan, 2016). Although national bilingual policy encourages active maintenance of mother tongues from the preschool years onward, actual home language practices have shifted substantially toward English; the 2020 Census reported that, for the first time, English (47.6%) surpassed Mandarin (40.2%) as the most frequently spoken home language among Chinese residents (Singapore Department of Statistics, 2021). Shared book reading is a setting in which this tendency is especially visible: parents choose which language to read in, how often to read, and how to guide the child during reading. These choices frequently diverge from parents’ stated language preferences, and the discrepancy between intended and actual mother-tongue practice has been documented across Singaporean households (Sun et al., 2022). Whether and how this gap between intention and practice relates to children’s bilingual development and to their socio-emotional functioning remains an open empirical question, with direct relevance for early childhood educators and policy makers in comparable multilingual contexts.

Two complementary perspectives help explain why bilingual shared reading decisions may matter for children’s development. The first concerns shared reading as language input. The input specificity hypothesis holds that children’s development in each language is shaped most directly by the amount and quality of input received in that language (Hoff, 2006; Place & Hoff, 2011). From this perspective, two aspects of home shared reading are especially relevant: how often parents read with children and how reading is allocated across languages. In Singapore, these decisions are consequential because English-medium schooling and public communication provide children with substantial English exposure outside the home, whereas Mandarin exposure depends much more heavily on family practices (Li & Tan, 2016; Sun, 2019). Home reading in Chinese is therefore not simply one literacy option among others, but a particularly important component of the home input on which Mandarin development depends for many Singaporean Chinese children. Research with dual-language learners further suggests that home language input typically shows language-specific associations rather than cross-language trade-offs: English proficiency does not necessarily require parental English use at home, whereas minority-language proficiency depends more strongly on support for that language in home and school contexts (Duursma et al., 2007), and bilingual children’s vocabulary and grammar in each language are related to the relative amount of input in that language (Hoff et al., 2012).

A second perspective concerns shared reading as a context for parent–child interaction. Shared book reading brings parents and children into sustained joint attention around a common text, creating opportunities for extended talk about characters, events, emotions, intentions, and interpretations (Fletcher & Reese, 2005; Schapira & Aram, 2020). From sociocultural and emotion-socialization perspectives, such adult-child interaction provides a scaffolded context in which adults guide children’s attention to social meanings and support their understanding of emotions, behaviors, and relationships (Eisenberg et al., 2010; Ng & Sun, 2022; Vygotsky, 1978). This interactional perspective points to a third dimension of home shared reading: parents’ reading orientation, defined here as their habitual emphasis in story talk—that is, what aspects of the story they direct children’s attention to and what kinds of meaning-making they prompt.

Prior work on mother–child storybook interaction has shown that parents may differ in whether they emphasize characters’ mental states and emotions or their external behaviors and actions (Doan & Wang, 2010). In the present study, we refer to parents’ emphasis on thoughts, feelings, and perspectives as cognitive orientation, and their emphasis on behaviors, rules, and consequences as behavioral orientation. This distinction is relevant to socio-emotional development because parent–child talk about emotions, intentions, behaviors, and consequences is one context in which adults socialize children’s understanding of emotions and social behavior (Eisenberg et al., 2010; Ng & Sun, 2022). In bilingual families, reading orientation is embedded within the same shared-reading practice in which parents also decide how much to read and which language to use, but it captures the interactional rather than input-related dimension of that practice.

This interactional dimension is particularly relevant in Singaporean Chinese families, where English and Mandarin are associated with socially differentiated functions rather than simple code choice. Prior Singapore research suggests that bilingual parents’ mental-state talk may shift with language context (Cheng et al., 2020), and that adult guidance during shared reading can shape children’s socio-emotional meaning-making in classroom settings (Ng & Sun, 2022). Although classroom evidence cannot be directly generalized to parent–child reading at home, these findings suggest that how adults guide children’s participation during shared reading is developmentally meaningful in the local context.

Children’s own language abilities are also relevant correlates of socio-emotional functioning, as language supports children’s ability to label feelings, take others’ perspectives, and manage social interactions (Clegg et al., 2015). In bilingual contexts, each language’s development may relate differently to socio-emotional outcomes (Sun et al., 2021b). The present analyses therefore consider children’s Chinese and English language abilities alongside parents’ reading practices in modeling socio-emotional outcomes.

Taken together, the present study distinguishes input-related dimensions of bilingual shared reading (reading quantity and language allocation) from its interactional dimension (reading orientation), while recognizing that these are embedded in the same home reading events. Examining them together therefore reflects a unified rationale: bilingual shared reading is simultaneously a language-input activity and a parent–child socialization context.

Existing research has rarely examined reading quantity, language allocation, and reading orientation alongside both language and socio-emotional outcomes within a single bilingual sample. Addressing this gap, the present study examines how these dimensions of home shared reading are associated with children’s Chinese and English language abilities and socio-emotional functioning in 39 Chinese-English bilingual preschoolers aged 5–6 years in Singapore. This age range captures the late preschool period before formal primary schooling, a developmental window in which Singaporean bilingual children show meaningful variation in language and socio-emotional outcomes (Sun, 2019; Sun et al., 2021b). Home input also remains relatively concentrated during this period (Hoff, 2006), providing a suitable context for examining family-level variation in bilingual home reading and child outcomes.

Three dimensions of shared reading—language allocation, reading quantity, and parents’ reading orientation—were examined in relation to children’s Chinese and English language abilities and to parent-reported socio-emotional functioning. Three research questions guide the analysis. RQ1 asks what patterns characterize Singapore bilingual shared book reading in terms of language allocation, reading quantity, and parents’ reading orientation. We expected reading to be more English-dominant in both language allocation and weekly reading time (H1a), and reported Chinese reading practices to fall short of parents’ stated mother-tongue intentions (H1b). RQ2 asks how these dimensions are associated with children’s Chinese and English language abilities. Following bilingual input-specificity accounts, we expected language-specific reading measures to be associated with children’s abilities in the corresponding language, with Chinese reading measures being especially relevant in Singapore’s English-dominant context (H2). RQ3 asks how these dimensions, together with children’s language abilities, are associated with prosocial and disruptive behaviors. We expected parents’ reading orientation to be associated with higher prosocial behavior and lower disruptive behavior after accounting for reading quantity and language allocation (H3a). Because children’s language abilities may also be associated with socio-emotional functioning, we further explored whether children’s Chinese and English language abilities, alongside Chinese and English weekly reading time, were associated with prosocial and disruptive behaviors (H3b). Given the modest sample size, findings are framed as associations rather than causal relationships.

## 2. Methods

### 2.1. Participants

This study employed a cross-sectional survey design to examine the relationships between bilingual shared book reading practices and children’s dual language and socio-emotional skills in Chinese families in Singapore. Data were collected through an online questionnaire administered during December 2025.

Participants were recruited through convenience and snowball sampling methods. Recruitment channels included local kindergartens and social media platforms. Participants were required to meet the following criteria: (a) parent or primary caregiver of a child aged 5–6 years; (b) child from a Chinese ethnic background; (c) child’s designated mother tongue language at school is Chinese (Mandarin); (d) family holds Singapore citizenship or permanent residency status; and (e) child has no parent-reported diagnosed developmental delays or disabilities that would significantly affect language or socio-emotional development.

A total of 43 responses were received. Four responses were excluded as not meeting inclusion criteria: three from families without Singapore citizenship or permanent residency status, and one from a non-Chinese ethnic background. The final sample consisted of 39 Chinese families. Among the respondents, 28 (71.8%) were mothers and 11 (28.2%) were fathers. Children’s mean age was 5.6 years (*SD* = 0.4), with 16 males (41.0%) and 23 females (59.0%). The sample was predominantly well-educated: 89.7% of mothers and 87.2% of fathers held at least a bachelor’s degree. The sample was also relatively affluent, with a median household income (SGD $14,500) exceeding the 2024 national median (SGD $11,297; Singapore Department of Statistics, 2025).

### 2.2. Measures

The research questions were examined using questionnaire data. Measures covered (a) child and family demographics, (b) home language profile, (c) home bilingual reading practices (quantity, allocation, and content/decision-making), (d) parents’ reading orientation, and (e) children’s socio-emotional behaviors. A bilingual (English–Chinese) questionnaire was administered; for each language-profile and reading-practice construct, the instrument contained parallel items for English and Chinese within the same survey. The full questionnaire is provided in Appendix A.

#### 2.2.1. Demographic Information

These variables included the child’s age, gender, and respondent’s relationship to the child. Family background variables included parents’ education levels and monthly household income. These variables were included as potential control variables in subsequent analyses.

#### 2.2.2. Language Profile

These measures were adapted from instruments used in prior Singapore bilingual research (Sun et al., 2018, 2023). Parents rated their own listening, speaking, reading, and writing proficiency in English and Chinese on 5-point scales (1 = not at all proficient to 5 = very proficient). For the present analyses, speaking and listening ratings were averaged within each language to form parent oral-proficiency composites, as these oral skills were most directly relevant to shared reading interaction. Child language abilities were assessed through parent ratings of the child’s speaking and understanding abilities in each language using a 5-point scale. Child language use was measured across six contexts (at school, at home, in parks/playgrounds, with friends, at family gatherings, and at public places) for both English and Chinese, using a 5-point frequency scale (1 = never to 5 = almost always). Parents also reported their psychological “first language” and the importance they placed on maintaining Chinese language exposure at home. Brief parent-rated ordinal scales of this type have been widely used in Singapore bilingual research (Sun, 2019; Sun et al., 2023), and prior validation work has supported caregiver/parent-report measures as useful indicators of bilingual children’s language exposure, use, and early abilities (Gutiérrez-Clellen & Kreiter, 2003; Marchman & Martínez-Sussmann, 2002).

#### 2.2.3. Home Bilingual Reading Practices

Reading practice measures were adapted from Sun (2019). Three dimensions were assessed separately for English and Chinese. Reading quantity included weekly shared reading frequency (days/week), daily duration (minutes/day), and library visit frequency. Resource allocation included the number of children’s books at home per language, the estimated percentage of reading time allocated to each language, and parents’ ideal allocation. When actual and ideal allocations differed, parents reported the main reasons for the discrepancy. Reading content included the types of books commonly read in each language and factors considered in book selection.

#### 2.2.4. Parents’ Reading Orientation

An 8-item Reading Interaction Style Scale was developed for this study, grounded in Bruner’s (1986) dual landscape theory and informed by Schapira and Aram’s (2020) application to shared reading. The scale assessed parents’ overall reported orientation toward shared reading and did not ask them to report separately on English and Chinese reading episodes. The scale comprised two subscales. The cognitive orientation subscale (4 items) assessed parents’ tendency to discuss characters’ feelings and thoughts, encourage predictions, connect stories to life experiences, and promote perspective-taking, drawing on emotion-talk coding systems from research with Chinese families (Curtis et al., 2020). The behavioral orientation subscale (4 items) assessed parents’ tendency to discuss right and wrong, check comprehension, discuss consequences, and teach moral lessons, informed by definitions of didactic talk in cross-cultural research (Wang et al., 2000). All items were rated on a 5-point scale (1 = never, 5 = always). The instrument demonstrated good internal consistency: cognitive orientation (*α* = 0.81), behavioral orientation (*α* = 0.84), and the overall reading orientation composite (*α* = 0.90).

#### 2.2.5. Children’s Socio-Emotional Behaviors

Socio-emotional behaviors were assessed using the Adaptive Social Behavior Inventory (ASBI; Hogan et al., 1992), a 30-item parent-report measure for preschool-aged children. Parents rated each behavior on a 3-point scale (1 = rarely, 2 = sometimes, 3 = almost always). The ASBI contains three item-level subscales: Express (13 items; social expressiveness), Comply (10 items; cooperation), and Disrupt (7 items; disruptive behavior). Following Hogan et al.’s (1992) scoring convention, the Express and Comply items were summed to form a composite Prosocial scale (23 items). The present analyses used the Prosocial composite and the Disrupt subscale as outcome variables. All scales demonstrated acceptable internal consistency: Express (*α* = 0.77), Comply (*α* = 0.82), Disrupt (*α* = 0.72), and Prosocial (*α* = 0.86).

### 2.3. Data Collection

Ethical approval was obtained from the Nanyang Technological University Institutional Review Board (IRB-2025-1038). The online questionnaire was hosted on Microsoft Forms and distributed through recruitment channels. Participants reviewed the study information sheet and provided electronic informed consent before proceeding. The questionnaire took approximately 10–15 min to complete. No compensation was provided.

### 2.4. Data Analysis

All statistical analyses were conducted using IBM SPSS Statistics Version 29. Prior to hypothesis testing, data were screened for missing values, outliers, and normality; no substantial missing data or outliers were identified. Internal consistency reliability of multi-item scales was assessed using Cronbach’s alpha.

To address Research Question 1 (patterns of bilingual reading practices), descriptive statistics (means, standard deviations, frequencies, and percentages) were computed. Paired-samples *t*-tests were used to compare English and Chinese measures, with Cohen’s d for paired samples calculated as the mean difference divided by the standard deviation of the difference scores.

Before conducting the regression analyses, bivariate correlations among study variables were examined. Several conceptually related variable pairs showed substantial correlations that would pose multicollinearity concerns for regression analyses, including the Chinese and English reading-frequency and allocation variables, the cognitive and behavioral orientation subscales, and within-language pairs of parental proficiency (speaking and listening) and children’s language abilities (speaking and understanding). The correlation between the cognitive and behavioral orientation subscales was particularly high (*r* = 0.80), which would introduce substantial multicollinearity if both were entered as simultaneous predictors. To address these issues, composite variables were constructed: A single SES factor was extracted from maternal education, paternal education, and household income, and Bartlett factor scores were used in subsequent analyses; Chinese and English weekly reading time (estimated minutes per week of language-specific shared reading), each computed as the product of reading days, daily reading time, and language-specific reading percentage; a reading orientation score averaging cognitive and behavioral orientation; and within-language averages for parental proficiency (speaking and listening) and children’s abilities (speaking and understanding). The weekly reading time composite was treated as a derived estimate of language-specific shared-reading dosage. Its construction drew on two related lines of work: studies that quantify home language activities using frequency and duration on a weekly basis (e.g., Florit et al., 2021), and bilingual exposure research showing that relative language exposure is a meaningful predictor of language outcomes (DeAnda et al., 2016; Hoff et al., 2012; Place & Hoff, 2011). Meta-analytic evidence further supports a documented association between home-based shared reading frequency and young children’s developmental outcomes (Galea et al., 2025).

To address Research Questions 2 and 3 (associations between reading practices and child outcomes), backward multiple regression analyses were used as an exploratory model-reduction procedure. For each of the four regression models, child gender was entered as a forced covariate in Block 1 to account for documented gender-related variation in early language and socio-emotional development (Bornstein et al., 2004; Sun et al., 2021b). Because gender was included as a background control rather than as a substantive predictor of interest, it was retained in all final models regardless of the backward-elimination procedure. The remaining a priori predictor set, defined separately for each outcome, was entered in Block 2 and subjected to backward elimination. Backward elimination was performed in SPSS Statistics 29 with a probability-of-*F*-to-remove criterion of 0.15, a relatively liberal threshold chosen to avoid overly aggressive variable deletion in this modest sample and close to the *p* ≈ 0.157 criterion corresponding to AIC-based selection for one-degree-of-freedom hierarchical comparisons (Heinze et al., 2018). Only the final retained model is reported.

The language-ability models included child gender as the forced covariate in Block 1 and four candidate predictors entered in Block 2 for backward elimination: SES factor score, language-specific weekly reading time, reading orientation, and language-specific parental proficiency. The Chinese language model is reported in the Results section, whereas the English language model is reported in text because the overall model did not reach statistical significance.

For the socio-emotional models, child gender was entered as the forced covariate in Block 1 and six candidate predictors entered in Block 2 for backward elimination: SES factor score, Chinese and English weekly reading time, reading orientation, and children’s Chinese and English language abilities. Chinese and English reading variables and child language abilities were entered into the initial model simultaneously because (1) the socio-emotional outcomes were not assessed in a language-specific manner, and (2) both language experience and language competence may be relevant to children’s socio-emotional wellbeing. Backward elimination then identified the most parsimonious subset of predictors retained in the final model. Parental language proficiency was not included in these models because the focus was on children’s own bilingual abilities and parents’ reading orientation as more proximal correlates of socio-emotional outcomes.

Regression diagnostics for the reported final models included variance inflation factors (VIF), residual plots, residual normality, and influential-case diagnostics. Multicollinearity and influential-case diagnostics did not indicate serious concerns; departures from residual normality for the English ability and Disrupt models are noted in the Limitations section. Post hoc power analyses for each final regression model were computed using SPSS Statistics 29 (Power Analysis: Linear Regression, Type III *F*-test) based on Cohen’s (1988) framework.

## 3. Results

### 3.1. Patterns of Bilingual Shared Book Reading Practices

#### 3.1.1. Language Profiles and Home Reading Practices

Table 1 presents the descriptive statistics for bilingual language profiles and home reading practices, with paired-samples *t*-tests comparing English and Chinese measures.

Based on parent self-report, parents rated their own English proficiency significantly higher (*M* = 4.28, *SD* = 0.84) than Chinese proficiency (*M* = 3.88, *SD* = 0.77). When asked about their psychological “first language” or “mother tongue,” the largest proportion of parents identified English (43.6%), followed by Chinese (28.2%) and both languages equally (28.2%). No parent identified a Chinese dialect as their primary language.

Parents rated children’s English abilities higher than their Chinese abilities across speaking, understanding, and language use measures. Parents rated their children’s English-speaking ability (*M* = 4.33, *SD* = 0.58) significantly higher than their Chinese speaking ability (*M* = 3.49, *SD* = 1.05), *p* < 0.001, *d* = 0.88. Similarly, English understanding (*M* = 4.38, *SD* = 0.59) was rated higher than Chinese understanding (*M* = 3.95, *SD* = 0.83), *p* < 0.001, *d* = 0.61. Children’s language use across contexts showed the largest disparity, with significantly more English use (*M* = 4.36, *SD* = 0.73) than Chinese use (*M* = 2.68, *SD* = 0.91), *p* < 0.001, *d* = 1.33.

Examining children’s language use across the six contexts, English use was consistently high across all six contexts, with means ranging from 4.15 to 4.54, whereas Chinese use was lower and more variable, with means ranging from 2.28 to 3.21. The English–Chinese gap was largest in peer-oriented contexts—in parks or playgrounds with peers (English *M* = 4.51, Chinese *M* = 2.28) and with friends (English *M* = 4.46, Chinese *M* = 2.36)—and smaller in family settings such as at home (English *M* = 4.21, Chinese *M* = 3.15) and at family gatherings (English *M* = 4.15, Chinese *M* = 2.62).

Home reading practices reflected this English-dominant pattern as well. Parents reported reading in English significantly more frequently (*M* = 3.37 days/week, *SD* = 1.77) than in Chinese (*M* = 1.72 days/week, *SD* = 1.76). Daily English reading time (*M* = 13.84 min, *SD* = 8.87) was nearly three times greater than Chinese reading time (*M* = 4.47 min, *SD* = 3.81). Families also possessed approximately twice as many English books (*M* = 59.78, *SD* = 45.69) as Chinese books (*M* = 29.21, *SD* = 29.14), *t*(38) = 5.76, *p* < 0.001, *d* = 0.92.

Despite the English-dominant language identity and practices, parents expressed strong positive attitudes toward maintaining Chinese for their children’s development. The vast majority (92.3%) rated Chinese language exposure at home as “very important” (51.2%) or “extremely important” (41.0%), with no parent rating it as unimportant (*M* = 4.33, *SD* = 0.62 on a 5-point scale).

Parents separately reported the percentage of total reading time they currently allocated to Chinese versus English (actual) and the percentage they would ideally allocate (ideal); the attitude-behavior gap was operationalized as the difference between these two (see Appendix A for questionnaire items). On average, English reading constituted 73.9% (*SD* = 18.14) of total reading time, with Chinese reading comprising only 26.1% on average (*SD* = 18.14). However, parents reported that their ideal allocation would be 55.3% English (*SD* = 9.39) and 44.7% Chinese (*SD* = 9.39). On average, parents aspired to provide 18.63 percentage points (*SD* = 17.54) more Chinese reading than they currently practiced. Of the 39 families, 24 (61.5%) reported that their ideal Chinese reading allocation exceeded their actual practice.

Parents who reported a discrepancy between their ideal and actual reading allocation were asked to identify the main reasons (multiple responses allowed). As shown in Table 2, the most frequently cited reason was limited time (66.7%), followed by the child’s preference for English (51.3%).

Language-related barriers differed substantially between Chinese and English. Parental language limitations were more commonly cited for Chinese (23.1%) than for English (2.6%); this pattern mirrors the proficiency differences shown in Table 1. Similarly, limited availability of suitable books was reported almost exclusively for Chinese (23.1%) compared to English (2.6%).

As shown in the upper panel of Table 3, the most cited factors for both languages were child-centered: child’s interests and preferences (English: 87.2%; Chinese: 76.9%) and content quality and educational value (English: 82.1%; Chinese: 61.5%). A similar proportion of parents considered the child’s language learning needs for both languages (English: 61.5%; Chinese: 64.1%).

However, an important language-specific pattern emerged for parents’ own language ability. This factor was cited by 23.1% of parents for Chinese book selection but only 2.6% for English.

The lower panel of Table 3 shows that stories and picture storybooks dominated in both languages equally (92.3%), and early reading/literacy books were also popular in both (English: 64.1%; Chinese: 56.4%). However, genre diversity differed markedly between the two languages. Science and educational books were far more commonly read in English (38.5%) than in Chinese (5.1%), a gap of over 33 percentage points. In contrast, traditional culture and history books were more commonly read in Chinese (10.3%) than in English (5.1%). Character-building/life skills books (English: 25.6%; Chinese: 17.9%) and emotional management/social skills books (English: 25.6%; Chinese: 20.5%) were used at moderately similar rates across languages, though with a slight English advantage.

#### 3.1.2. Reading Orientations

Table 4 presents the descriptive statistics for parental reading orientation during shared book reading. Parents demonstrated significantly higher behavioral orientation (*M* = 3.42, *SD* = 0.76) than cognitive orientation (*M* = 3.04, *SD* = 0.84) during shared reading, *t*(38) = 4.66, *p* < 0.001, *d* = 0.75. Within behavioral orientation, the most frequently reported behaviors were teaching moral lessons (*M* = 3.56, *SD* = 0.82) and discussing whether characters’ behaviors were right or wrong (*M* = 3.51, *SD* = 0.94). Within cognitive orientation, connecting stories to children’s life experiences was most common (*M* = 3.33, *SD* = 1.03), while encouraging children to imagine being a character was least frequent (*M* = 2.74, *SD* = 1.07).

The following sections report how these reading dimensions were associated with children’s dual language and socio-emotional outcomes.

### 3.2. Home Reading Practices and Children’s Dual Language Abilities

Table 5 presents the bivariate correlations among study variables, which informed the construction of the composite variables described in Section 2.4.

In the Chinese language model, child gender was entered as a forced covariate. The final retained model included Chinese weekly reading time and parental Chinese proficiency in addition to the gender covariate. The overall model approached but did not reach conventional statistical significance, *F*(3, 35) = 2.67, *p* = 0.063, *R*^2^ = 0.19. Within this model, Chinese weekly reading time, *β* = 0.333, *p* = 0.038, and parental Chinese proficiency, *β* = 0.318, *p* = 0.049, were positively associated with children’s Chinese language ability. These coefficient-level associations should be interpreted cautiously given the marginal omnibus test, as shown in Table 6.

For children’s English language ability, the overall model did not reach statistical significance, *F*(2, 36) = 1.42, *p* = 0.256, *R*^2^ = 0.07. Parental English proficiency was retained but did not reach significance (*β* = 0.246, *p* = 0.141). English weekly reading time was not retained by backward selection.

### 3.3. Home Reading Practices and Children’s Socio-Emotional Behaviors

The initial models for ASBI Prosocial and ASBI Disrupt each included child gender, SES factor score, Chinese weekly reading time, English weekly reading time, reading orientation, and children’s Chinese and English language abilities.

For prosocial behavior, the final model was statistically significant, *F*(3, 35) = 9.37, *p* < 0.001, *R*^2^ = 0.45. Reading orientation, *β* = 0.385, *p* = 0.004, and children’s Chinese language ability, *β* = 0.470, *p* < 0.001, were positively associated with prosocial behavior, with child gender entered as a forced covariate. The final model is presented in Table 7.

For disruptive behavior, the overall model did not reach statistical significance, *F*(2, 36) = 2.64, *p* = 0.085, *R*^2^ = 0.13.

## 4. Discussion

The present study examined bilingual shared book reading practices among Chinese families in Singapore and their associations with children’s dual language learning and socio-emotional skills. Three sets of findings emerged. First, families in this sample displayed an English-dominant reading pattern alongside a substantial attitude–behavior gap in Mandarin reading, and reported stronger behavioral than cognitive reading orientation during shared reading. Second, exploratory backward regression analyses suggested differentiated patterns across reading dimensions and languages: Chinese weekly reading time and parental Chinese proficiency were positively associated with children’s Chinese language ability, although the overall model approached but did not reach conventional significance, whereas the English language model did not reach significance and English weekly reading time was not retained. Third, children’s Chinese language ability and parents’ reading orientation were positively associated with prosocial behavior, whereas the disruptive behavior model did not reach significance. Taken together, the present results align with the dual framing introduced in the Introduction: input-related variables (Chinese reading time and parental Chinese proficiency) showed positive associations with children’s Chinese language ability, while the interaction-related dimension of reading orientation, together with children’s Chinese language ability, was associated with prosocial behavior. These patterns do not establish causal direction or underlying mechanisms and warrant replication in larger, longitudinal samples.

### 4.1. Bilingual Reading Practices and the Attitude-Behavior Gap

The descriptive findings can be read as a three-layer portrait of home bilingual shared reading in this sample: a structural layer in which English dominates across the language profile and reading practices; a psychological layer marked by the attitude-behavior gap between parents’ Mandarin aspirations and their actual practice; and an adaptive layer characterized by a more behavioral than cognitive reading orientation when reading did occur. We discuss each layer in turn below.

*The structural layer: English dominance across domains.* English dominated families’ shared book reading practices, with English constituting 73.9% of total reading time compared to 26.1% for Chinese. This English dominance extended beyond reading: children’s language use across six social contexts showed a large English-Chinese gap (*d* = 1.33), and parents’ ratings of children’s English-speaking ability were substantially higher than their Chinese-speaking ability (*d* = 0.88). The English–Chinese gap was particularly pronounced in peer-oriented settings such as playgrounds and with friends, where English overwhelmingly predominated, compared to relatively smaller gaps in family contexts such as at home and at family gatherings. This context-dependent pattern is consistent with Sun et al.’s (2022) finding that the majority of 201 Singaporean kindergarteners used Mandarin and English interchangeably at home and school but predominantly spoke English in the wider community—at playgrounds, while shopping, and at dining places—leaving little chance for Mandarin to be used. The findings reflect the broader language shift documented in Singapore, where English has increasingly become the dominant home language (Cavallaro & Ng, 2014; Singapore Department of Statistics, 2021).

*The psychological tension: from valued aspiration to lived practice.* Despite English dominance in practice, 92.3% of parents rated Chinese exposure as “very” or “extremely” important, and their ideal Chinese reading allocation (44.7%) substantially exceeded actual practice (26.1%). This attitude-behavior gap is substantial: despite strong positive attitudes toward mother tongue maintenance, families clearly face practical challenges in translating these beliefs into actual reading practices. This 18-percentage-point gap is consistent with Family Language Policy research documenting that positive attitudes toward mother tongue often do not translate into practice (Curdt-Christiansen, 2016; Liang et al., 2022). Parents cited limited time (66.7%) and children’s preference for English (51.3%) as primary barriers. Notably, 23.1% of parents also identified their own limited Chinese proficiency as a constraint, underscoring how parental language limitations specifically compound the challenges of mother tongue reading (Sun et al., 2023).

The reported barriers showed a language-specific pattern: parental language limitations and book availability were cited predominantly as constraints for Chinese reading. In this sample, the barriers endorsed by parents were more often family-level—parental time, children’s language preference, parents’ own language limitations, and unequal access to reading materials—than school-related.

*Language identity and functional differentiation.* Underlying this gap is a notable shift in parental language identity. Although all participants were ethnic Chinese, 43.6% identified English as their first language, compared to only 28.2% for Chinese. This creates a paradox: parents who themselves identify primarily with English nonetheless value Chinese for their children, possibly reflecting the socially differentiated functions often attributed to the two languages in Singapore—English for instrumental advancement and Chinese for cultural identity and family cohesion (Curdt-Christiansen, 2016). The genre patterns in shared reading further support this functional differentiation: traditional culture and history books were more commonly read in Chinese (10.3%) than in English (5.1%), whereas science and educational books were far more prevalent in English (38.5% vs. 5.1%), suggesting that parents may purposefully use Chinese reading as a vehicle for cultural transmission even when English dominates overall reading volume. Parents with limited Chinese proficiency may recognize the importance of mother tongue reading but face practical barriers to providing it—a tension that may partly explain why the attitude-behavior gap persists even among families with strong pro-bilingual beliefs.

### 4.2. Language-Specific Associations Between Home Reading and Dual Language Abilities

The regression analyses suggested differentiated patterns across the Chinese and English language models. In the Chinese language model, Chinese weekly reading time and parental Chinese proficiency showed positive associations with children’s Chinese language ability, although the overall model approached but did not reach conventional significance. In the English language model, by contrast, the overall model did not reach significance, and English weekly reading time was not retained. Because these findings differed across the two language models, they are discussed separately below.

The Chinese language findings are broadly consistent with input-specificity accounts and prior research on home literacy environments. Shared reading provides language-specific input, and prior studies have shown that home literacy practices are associated with children’s language development (Bus et al., 1995; Mol & Bus, 2011), including in bilingual populations (Duursma et al., 2007; Goodrich et al., 2021). In Singapore specifically, Sun (2019) found that the home Mandarin language and literacy environment was associated with bilingual preschoolers’ receptive Mandarin proficiency, and Li and Tan (2016) showed that home literacy activities were associated with Singaporean bilingual children’s Chinese oral and written language abilities. In the present study, the Chinese language model retained both Chinese weekly reading time and parental Chinese proficiency, suggesting that children’s Chinese language ability may be related not only to the amount of Chinese shared-reading input but also to parents’ own mother-tongue resources. This interpretation is consistent with Sun et al. (2023), who showed that parental language resources shape home language practices, and with the harmonious bilingual experience framework, which emphasizes the interconnection between parental language resources and child outcomes (Sun, 2023). Given that the overall Chinese model was marginal, however, these associations should be interpreted as preliminary and warrant replication in larger samples.

The non-significant English model should also be interpreted cautiously. English weekly reading time was not retained, and the overall model did not reach significance. One possible explanation, consistent with the relative weight hypothesis (Sun et al., 2021a), is that when children already receive abundant input in a societal language, additional home reading in that language may account for less detectable variance in language outcomes than home reading in a less societally supported language. In Singapore, where children receive substantial English exposure through school and broader social environments, English outcomes may be supported by multiple input sources beyond home shared reading. This interpretation is also consistent with recent Singapore evidence from a different domain of additional language exposure: extra-curricular English classes were not significantly associated with children’s English receptive vocabulary or word-reading, whereas Mandarin enrichment classes were positively associated with Mandarin word-reading (Sun et al., 2026). However, this interpretation remains tentative. A ceiling effect in parent-rated English ability, together with the low power of the English-ability model, may also have contributed to the null finding. Thus, the present data are compatible with—but do not establish—the possibility that home reading associations are more detectable for mother-tongue outcomes than for English outcomes in this setting. Larger samples with greater variability in English reading engagement are needed to determine whether this pattern reflects substantive developmental processes or sampling and measurement constraints.

### 4.3. Reading Orientation, Children’s Chinese Ability, and Prosocial Behavior

The prosocial behavior model provided the clearest socio-emotional finding in this study. Reading orientation and children’s Chinese language ability were positively associated with prosocial behavior, with child gender included as a forced covariate. This pattern provides partial support for H3a: parents’ reading orientation was associated with prosocial behavior, although it was not retained in the disruptive behavior model. Under H3b, children’s Chinese language ability was also associated with prosocial behavior, whereas Chinese reading quantity was not retained in the prosocial model.

Children’s Chinese language ability showed the larger standardized coefficient in the prosocial model (*β* = 0.470, *p* < 0.001). Because this association emerged from the exploratory H3b analysis rather than from an a priori prediction, it should be treated as a tentative association rather than as an established finding. The finding is compatible with broader evidence linking language skills and social competence: Wieczorek et al. (2025) reported a medium-sized association between language abilities and social competence, and Sun et al. (2021b) found that larger bilingual receptive vocabulary and more frequent bilingual output were associated with better socio-emotional skills among Singaporean preschoolers. Whether the present association reflects mother-tongue-specific processes, language ability more generally, or correlated features of family experience cannot be determined from the present cross-sectional data.

Reading orientation was also positively associated with prosocial behavior. This suggests that parents’ reported ways of guiding children through stories may be relevant to prosocial outcomes beyond reading quantity and language allocation. Descriptively, parents reported higher behavioral than cognitive orientation, with moral-lesson talk and discussion of right and wrong more strongly endorsed than perspective-taking prompts. This pattern resonates, at a descriptive level, with classroom-based observations from Singapore, where teachers more often supported interpersonal than intrapersonal learning during shared book reading (Ng & Sun, 2022). The present findings do not establish why this pattern appears, but they suggest that reading orientation is a relevant dimension of home shared reading when considering prosocial outcomes in this sample.

In contrast, the disruptive behavior model did not reach statistical significance and was not interpreted as evidence of a reliable association. This null pattern should be interpreted cautiously given the modest power of the Disrupt regression. It may also reflect that disruptive behavior is shaped by self-regulatory and temperamental factors not captured by the present reading-practice measures (Eisenberg et al., 2010). Larger and more behaviorally variable samples will be needed to determine whether any component of home shared reading is associated with disruptive behavior.

### 4.4. Integrating the Findings: Patterns and Boundaries

Viewed together, the findings point to a differentiated but limited pattern of associations rather than to a single explanatory mechanism. Chinese weekly reading time and parental Chinese proficiency were positively associated with children’s Chinese language ability, whereas reading orientation and children’s Chinese language ability were associated with prosocial behavior. These patterns are consistent with the dual framing of shared reading as both an input-related and interactional context, but they remain preliminary given the small sample, cross-sectional design, and exploratory model-selection approach.

### 4.5. Limitations and Future Directions

Several limitations should be considered when interpreting the findings of this study. First, the sample size was relatively small (*N* = 39), which limited statistical power and the stability of regression estimates. The regression analyses used backward selection as an exploratory model-reduction approach, and the retained variables should therefore be interpreted as candidate associations rather than as confirmatory evidence for a pre-specified model. Post hoc power analyses based on the final retained models indicated high observed power for the Prosocial model (1 − *β* = 0.997), whereas power was below the conventional 0.80 threshold for the Chinese ability (1 − *β* = 0.652), Disrupt (1 − *β* = 0.526), and English ability models (1 − *β* = 0.305). The marginal Chinese ability model and the null findings for the English ability and Disrupt models should therefore be interpreted cautiously, as Type II error cannot be ruled out. Replication with larger samples is needed to assess the stability and generalizability of these patterns.

Second, the cross-sectional design precludes causal inference. The associations observed between reading practices, language ability, and prosocial behavior may reflect direct associations, shared underlying family factors, or bidirectional processes. Future longitudinal research is needed to examine how home reading practices, children’s bilingual abilities, and socio-emotional development unfold over time.

Third, all measures were based on parent self-report, which may be subject to social desirability bias and recall limitations. Parents may overestimate their reading frequency or their children’s abilities. Although parent-report measures are useful indicators of bilingual children’s language exposure, use, and early abilities, they cannot replace direct child language assessments. Future studies should include direct language assessments where feasible to validate and extend the present parent-reported findings. Additionally, the barrier items used a fixed-option checklist format, which may have shaped which barriers parents endorsed and under-represented barriers not listed among the response options. Future research could incorporate objective measures such as reading diaries, home observations of parent–child reading interaction, open-ended barrier questions, or standardized assessments of children’s language and socio-emotional abilities.

Fourth, reading orientation was assessed as an overall parent-reported characterization rather than separately within English and Chinese reading episodes. The present study therefore cannot determine whether parents’ cognitive or behavioral orientation differs across languages within the same family. Future research using language-specific orientation measures or observations of actual English and Chinese reading episodes could examine this question at a finer level of analysis.

Fifth, residual diagnostics indicated departures from normality for the English ability and Disrupt models, whereas the Chinese ability and Prosocial models did not show the same concern. This provides an additional reason for interpreting the English and Disrupt models cautiously.

Sixth, cognitive and behavioral orientation were descriptively distinguishable but highly correlated in this sample (*r* = 0.80). To avoid multicollinearity, the regression analyses used an overall reading orientation composite. The present analyses therefore cannot determine whether the two subscales have distinct associations with socio-emotional outcomes. Future studies with larger samples or observational coding could examine whether different features of reading orientation make separable contributions to children’s socio-emotional development.

Finally, the sample was predominantly mothers, highly educated, relatively affluent, and limited to Chinese families in Singapore. This limits the generalizability of findings to the broader population of Singaporean Chinese families and to other bilingual populations. Families with different socioeconomic profiles or linguistic backgrounds may face different barriers and demonstrate different patterns of bilingual reading practices. Future research should examine whether the patterns observed here—particularly the associations among mother-tongue reading, parental mother-tongue proficiency, children’s mother-tongue ability, and prosocial behavior—generalize across diverse bilingual populations and linguistic contexts.

## 5. Conclusions

This study examined how reading quantity, language allocation, and reading orientation in home shared book reading relate to bilingual children’s language abilities and socio-emotional behaviors among 39 Chinese families in Singapore. Three findings stand out. First, families in this sample showed a pronounced English-dominant reading pattern alongside a substantial attitude–behavior gap, despite overwhelmingly positive attitudes toward Chinese exposure. Second, Chinese weekly reading time and parental Chinese proficiency were positively associated with children’s Chinese language ability, although the overall Chinese model was marginal; by contrast, the English language model did not reach significance, and English weekly reading time was not retained. Third, children’s Chinese language ability and parental reading orientation were positively associated with prosocial behavior, whereas the disruptive behavior model did not provide evidence of a reliable association. Taken together, these findings suggest that home shared reading may be relevant to bilingual children’s language and socio-emotional development through both input-related and interaction-related dimensions.

For families navigating bilingual reading decisions, the present findings—though preliminary—tentatively suggest that regular mother-tongue shared reading at home, alongside parents’ ways of guiding children during reading, may be relevant to children’s Chinese language ability and prosocial behavior. Practical implications should be drawn cautiously, particularly given the marginal nature of the Chinese language model and the cross-sectional design. These associations require replication in larger, longitudinal samples.

## Figures and Tables

**Table 1:** Bilingual Language Profiles and Home Reading Practices (*N* = 39).

Variable	English	Chinese	*t*	*p*
**Parent language proficiency**				
Overall (1–5 scale)	4.28 (0.84)	3.88 (0.77)	2.19	0.034
**Child language proficiency**				
Speaking (1–5 scale)	4.33 (0.58)	3.49 (1.05)	5.50	<0.001
Understanding (1–5 scale)	4.38 (0.59)	3.95 (0.83)	3.79	<0.001
**Child language use**				
Overall (1–5 scale)	4.36 (0.73)	2.68 (0.91)	8.32	<0.001
**Home reading practices**				
Reading frequency (days/week)	3.37 (1.77)	1.72 (1.76)	6.86	<0.001
Reading time (min/day)	13.84 (8.87)	4.47 (3.81)	5.89	<0.001
Number of books at home	59.78 (45.69)	29.21 (29.14)	5.76	<0.001
Actual reading allocation (%)	73.88(18.14)	26.12(18.14)	8.35	<0.001
Ideal reading allocation (%)	55.26 (9.39)	44.74 (9.39)	3.50	0.001

*Note*. Standard deviations are presented in parentheses. Parent language proficiency was rated on a 5-point scale (1 = no fluency, 5 = very fluent). Child speaking ability was rated on a 5-point scale (1 = speech so halting that conversation is virtually impossible, 5 = fluent and effortless). Child language understanding was rated on a 5-point scale (1 = cannot understand simple conversation, 5 = understands everyday conversation and normal discussion). Child language use was rated on a 5-point frequency scale (1 = never, 5 = almost always).

**Table 2:** Reasons for Attitude-Behavior Gap in Reading Allocation (*N* = 39).

Reason	*n*	%
Limited total time for parent(s)	26	66.7
Child’s language preference (e.g., prefers English)	20	51.3
Limited availability of suitable Chinese books	9	23.1
Parent’s own limited Chinese proficiency	9	23.1
Limited availability of suitable English books	1	2.6
Parent’s own limited English proficiency	1	2.6
School requirements or expectations	2	5.1
Other	2	5.1

*Note*. Multiple responses allowed.

**Table 3:** Book Selection Factors and Types of Books Read.

	English		Chinese	
	*n*	%	*n*	%
**Factors considered**				
Child’s interests and preferences	34	87.2	30	76.9
Content quality and educational value	32	82.1	24	61.5
Child’s language learning needs	24	61.5	25	64.1
Parent’s own language ability	1	2.6	9	23.1
Book availability	7	17.9	7	17.9
**Types of books**				
Stories/picture storybooks	36	92.3	36	92.3
Early reading/literacy books	25	64.1	22	56.4
Science/educational books	15	38.5	2	5.1
Traditional culture/history	2	5.1	4	10.3
Character-building/life skills	10	25.6	7	17.9
Emotional management/social skills	10	25.6	8	20.5

*Note*. Multiple responses allowed.

**Table 4:** Reading Orientations (*N* = 39).

Variable	*M*	*SD*
**Cognitive orientation**		
Discuss feelings and thoughts	3.21	1.00
Encourage predictions	2.87	1.08
Connect to life experience	3.33	1.03
Imagine as character	2.74	1.07
Subscale mean	3.04	0.84
**Behavioral orientation**		
Discuss right/wrong	3.51	0.94
Check understanding	3.36	1.06
Discuss consequences	3.26	0.82
Teach moral lessons	3.56	0.82
Subscale mean	3.42	0.76

*Note*. Items rated on a 1–5 scale (1 = never, 5 = always).

**Table 5:** Pearson Bivariate Correlations among Study Variables.

Variable	1	2	3	4	5	6	7	8	9	10	11	12	13	14	15	16	17	18	19	20
1. Child gender	—																			
2. Mother education	−0.17	—																		
3. Father education	−0.25	0.27	—																	
4. Family income	−0.03	0.13	0.28	—																
5. Chi read days	−0.17	−0.03	0.15	0.26	—															
6. Eng read days	0.04	−0.06	0.09	0.41 **	0.64 **	—														
7. Daily read time	0.32 *	−0.04	0.18	0.21	−0.27	0.05	—													
8. Reading Chi %	0.07	−0.06	0.09	−0.01	0.37 *	−0.07	−0.23	—												
9. Reading Eng %	0.02	0.11	−0.10	−0.06	−0.25	0.11	0.14	−0.91 **	—											
10. Cognitive orientation	−0.02	−0.18	0.10	0.18	0.09	0.03	−0.15	0.18	−0.11	—										
11. Behavioral orientation	−0.05	−0.23	−0.01	0.28	0.17	0.24	−0.06	−0.08	0.10	0.80 **	—									
12. Parent Chi speak	−0.13	0.11	0.04	0.07	0.10	−0.04	−0.16	0.14	−0.09	0.49 **	0.33 *	—								
13. Parent Chi listen	−0.18	0.14	0.06	0.09	0.03	−0.04	−0.05	−0.02	0.03	0.40 *	0.23	0.79 **	—							
14. Parent Eng speak	−0.16	0.02	0.09	0.12	−0.07	0.17	0.06	−0.32 *	0.28	0.30	0.38 *	0.08	0.29	—						
15. Parent Eng listen	−0.18	0.00	−0.04	0.06	−0.04	0.18	0.05	−0.38 *	0.39 *	0.16	0.32	0.00	0.27	0.93 **	—					
16. Child Chi speak	0.09	−0.14	0.25	−0.02	0.43 **	0.28	−0.22	0.40 *	−0.28	0.02	−0.07	0.13	0.08	−0.36 *	−0.32 *	—				
17. Child Chi understand	0.01	−0.14	0.02	0.05	0.26	0.06	−0.10	0.37 *	−0.26	0.10	0.01	0.40 *	0.32 *	−0.16	−0.17	0.61 **	—			
18. Child Eng speak	−0.15	0.10	0.25	0.06	0.24	0.25	−0.20	−0.05	0.09	0.04	0.00	0.11	0.23	0.24	0.17	0.42 **	0.48 **	—		
19. Child Eng understand	−0.08	0.08	0.22	−0.07	−0.10	−0.04	0.00	−0.15	0.18	−0.03	−0.11	0.17	0.31	0.31	0.23	0.20	0.53 **	0.77 **	—	
20. ASBI Prosocial	0.27	−0.21	0.07	0.02	0.26	0.13	0.07	0.05	0.10	0.36 *	0.37 *	0.08	−0.01	−0.05	−0.08	0.46 **	0.42 **	0.21	0.18	—
21. ASBI Disrupt	−0.17	0.09	0.14	−0.02	0.43 **	0.16	−0.15	0.22	0.01	0.23	0.20	−0.02	−0.01	0.06	0.08	0.29	0.25	0.22	0.15	0.31

*Note*. * *p* < 0.05. ** *p* < 0.01. Chi = Chinese; Eng = English.

**Table 6:** Final Backward Regression Model: Child Chinese Ability.

Variable	B	SE	*β*	*t*	*p*
Child gender	0.146	0.261	0.086	0.557	0.581
Chi weekly read time	0.029	0.013	0.333	2.158	0.038
Parent Chi proficiency	0.386	0.189	0.318	2.039	0.049

*Note*. *R*^2^ = 0.186, Adj. *R*^2^ = 0.116. *F*(3, 35) = 2.669, *p* = 0.063. Chi = Chinese.

**Table 7:** Final Backward Regression Model: ASBI Prosocial Behavior.

Variable	B	SE	*β*	*t*	*p*
Child gender	2.823	1.411	0.252	2.000	0.053
Reading orientation	2.836	0.927	0.385	3.057	0.004
Child Chi ability	3.113	0.835	0.470	3.727	<0.001

*Note*. *R*^2^ = 0.445, Adj. *R*^2^ = 0.398. *F*(3, 35) = 9.373, *p* < 0.001. Chi = Chinese.

## Data Availability

The data presented in this study are available upon request from the corresponding author. Restrictions in data availability are due to privacy and ethical reasons.

## Appendix A

### Appendix A.1. Home Bilingual Reading and Language Survey

**Note on language versions.** The questionnaire was administered as a bilingual instrument, with parallel English and Mandarin Chinese versions for each item presented in the same survey. For space reasons, only the English version is reproduced below; the Chinese version was a direct translation reviewed by bilingual researchers and is available from the corresponding author upon request.

### Appendix A.2. Part 1: Child and Family Demographics

#### Appendix A.2.1. Section A: Child Information

1. Your child’s date of birth (DD/MM/YYYY): ___________
2. Your child’s gender: □ Male □ Female
3. Your relationship to the child:
  □ Father
  □ Mother
  □ Other (please specify: ___________)
4. What Mother Tongue Language is the child learning in school?
  □ Mandarin Chinese
  □ Malay
  □ Tamil
  □ Others (please specify): ___________
5. Has your child been diagnosed with any learning or developmental issues?
  □ No
  □ Yes (please provide further information: _________)
6. Do you have any concerns regarding your child’s learning development? (e.g., reading, math, speaking, behavior regulation, emotional regulation)
  □ No
  □ Yes (please provide further information: _________)

#### Appendix A.2.2. Section B: Family Language Background

7. Please rate YOUR proficiency in ENGLISH:

**Skill**	**1**	**2**	**3**	**4**	**5**
Listening					
Speaking					
Reading					
Writing					
*Note*. 1 = No fluency (no understanding or speaking ability); 2 = Limited fluency (some understanding, can say short simple sentences); 3 = Somewhat fluent (good understanding, can express on many topics); 4 = Quite fluent (can understand and use language adequately for work and most situations); 5 = Very fluent (understands almost everything, very comfortable expressing in all situations).					

8. Please rate YOUR proficiency in CHINESE:

**Skill**	**1**	**2**	**3**	**4**	**5**
Listening					
Speaking					
Reading					
Writing					
*Note*. 1 = No fluency (no understanding or speaking ability); 2 = Limited fluency (some understanding, can say short simple sentences); 3 = Somewhat fluent (good understanding, can express on many topics); 4 = Quite fluent (can understand and use language adequately for work and most situations); 5 = Very fluent (understands almost everything, very comfortable expressing in all situations).					

9. How well does YOUR CHILD SPEAK in English and Chinese?

**Language**	**1**	**2**	**3**	**4**	**5**
English					
Chinese					
*Note*. 1 = Speech so halting and fragmented that conversation is virtually impossible; 2 = Usually hesitant, often forced into silence by language limitations; 3 = Speech in everyday conversation frequently disrupted by search for the correct manner of expression; 4 = Speech generally fluent with occasional lapses while searching for the correct manner of expression; 5 = Speech fluent and effortless, approximating that of a native speaker.					

10. How well does YOUR CHILD UNDERSTAND spoken English and Chinese?

**Language**	**1**	**2**	**3**	**4**	**5**
English					
Chinese					
*Note*. 1 = Cannot be said to understand even simple conversation; 2 = Has great difficulty following what is said, can only comprehend social conversation spoken slowly and with frequent repetitions; 3 = Understands most of what is said at slower-than-normal speed with repetitions; 4 = Understands nearly everything at normal speech, occasional repetition may be necessary; 5 = Understands everyday conversation and normal classroom discussions.					

11. Please estimate how often your child uses Chinese and English in the following situations (1 = Never, 2 = Rarely, 3 = Sometimes, 4 = Often, 5 = Almost Always):

**In This Situation…**	**English (1–5)**	**Chinese (1–5)**
At school (with teachers/classmates)	1 2 3 4 5	1 2 3 4 5
At home (with family members)	1 2 3 4 5	1 2 3 4 5
In a park or playground (with peers)	1 2 3 4 5	1 2 3 4 5
In the company of friends	1 2 3 4 5	1 2 3 4 5
At a party with extended family	1 2 3 4 5	1 2 3 4 5
At a shopping mall or restaurant	1 2 3 4 5	1 2 3 4 5

12. At home, what language do you primarily use to communicate with your child?
  □ Almost exclusively English
  □ Mainly English with some Chinese
  □ About equal amounts of English and Chinese
  □ Mainly Chinese with some English
  □ Almost exclusively Chinese
13. How important is it for your child to maintain Chinese language exposure at home?
  □ 1—Not important at all
  □ 2—Slightly important
  □ 3—Moderately important
  □ 4—Very important
  □ 5—Extremely important
14. Psychologically, what do you consider your “first language” or “mother tongue”?
  □ Chinese
  □ English
  □ Both Chinese and English
  □ Dialect (please specify: ___________)
  □ Other (please specify: ___________)
15. Which language do you feel most comfortable using to express emotions?
  □ Chinese
  □ English
  □ Both equally
  □ Dialect (please specify: ___________)
  □ Other (please specify: ___________)

### Appendix A.3. Part 2: Home Bilingual Reading Practices

#### Appendix A.3.1. Reading Frequency and Duration

16. Who primarily reads with your child at home? (Select all that apply)
  □ Mother
  □ Father
  □ Grandparent(s)
  □ Domestic helper
  □ Older sibling(s)
  □ Others (please specify: ___________)
17. How many days per week does someone read to your child? (0 = No reading, 1–7 = days per week)

	**0**	**1**	**2**	**3**	**4**	**5**	**6**	**7**
English books								
Chinese books								
Other books (specify): ___________								

18. How many days per week does your child read on their own?

	**0**	**1**	**2**	**3**	**4**	**5**	**6**	**7**
English books								
Chinese books								
Other books (specify): ___________								

19. On reading days, approximately how many TOTAL minutes per day?
  □ 0 min
  □ Less than 15 min
  □ 15–30 min
  □ 31–45 min
  □ 46–60 min
  □ More than 60 min
20. How often does your child go to the library?
  □ Never
  □ Once a month
  □ Once every 2 weeks
  □ Once a week
  □ Twice a week
  □ More than twice a week

#### Appendix A.3.2. Section B: English-Chinese Reading Ratio and Resources

21. When different family members read with your child, what language do they typically use?

**Family Member**	**Mainly Chinese**	**Equal Mix**	**Mainly English**	**Does not Read with Child**
Mother	□	□	□	□
Father	□	□	□	□
Grandparent(s)	□	□	□	□
Domestic helper	□	□	□	□
Older sibling(s)	□	□	□	□
Others	□	□	□	□

22. Of your total reading time, what percentage is in each language? (Must add to 100%)
  Chinese books: ______% English books: ______%
23. How many children’s books do you have at home?

**Number of Books**	**Chinese Books**	**English Books**
None	□	□
1–10	□	□
11–30	□	□
31–60	□	□
61–90	□	□
91–120	□	□
More than 120	□	□

24. What format of books do you primarily use for shared reading?

**Book Format**	**Chinese Books**	**English Books**
Exclusively physical books	□	□
Mainly physical books	□	□
About equal mix of physical and digital	□	□
Mainly e-books/digital books	□	□
Exclusively e-books/digital books	□	□

#### Appendix A.3.3. Section C: Reading Content and Decision-Making

25. What types of books do you most commonly read with your child? (Select all that apply for each language)

**Book Types**	**Chinese Books**	**English Books**
Stories/Picture books	□	□
Language learning/Literacy	□	□
Science/Educational	□	□
Traditional culture/History	□	□
Character building/Life skills	□	□
Emotional management/Social skills	□	□
Other (please specify): ___________	□	□

26. When selecting books, which factors do you primarily consider? (Select up to 3 for each language)

**Selection Factors**	**Chinese Books**	**English Books**
Child’s interests and preferences	□	□
Child’s language learning needs	□	□
Content quality and educational value	□	□
My own language ability and reading comfort	□	□
Cultural identity preservation	□	□
School teacher requirements or recommendations	□	□
Book availability and accessibility	□	□
Other (please specify): ___________	□	□

27. Please list three of your child’s current favorite CHINESE books:
28. Please list three of your child’s current favorite ENGLISH books:
29. Ideally, what would be your preferred reading ratio? (Must add to 100%)
  Ideal Chinese reading: ______% Ideal English reading: ______%
30. If your current practice differs from your ideal, what are the main reasons? (Select all that apply)
  □ Limited total time for me and my spouse
  □ Limited availability of suitable Chinese books
  □ Limited availability of suitable English books
  □ Child’s language preference
  □ My own limited Chinese proficiency
  □ My own limited English proficiency
  □ School requirements/expectations
  □ Other (please specify: ___________)

#### Appendix A.3.4. Section D: Reading Interaction Style

Please rate how often you use these approaches during shared reading

(1 = Never, 2 = Rarely, 3 = Sometimes, 4 = Often, 5 = Always):

**During Shared Reading, I Usually…**	1	2	3	4	5
31. Discuss characters’ feelings and thoughts (e.g., “How do you think the little pig felt when the wolf huffed and puffed?”)					
32. Encourage my child to make predictions about the story (e.g., “What do you think will happen when the wolf comes?”)					
33. Connect story events to my child’s own life experiences (e.g., “Remember when you worked hard to build that tall tower with blocks?”)					
34. Encourage perspective-taking by asking my child to imagine being a character (e.g., “What would you do if you were the little pig?”)					
35. Discuss whether characters’ behaviors are right or wrong (e.g., “Was it a good idea for the first pig to build his house so quickly?”)					
36. Ask questions to check story comprehension (e.g., “What happened to the straw house? Where did the pigs run to?”)					
37. Discuss consequences of characters’ actions (e.g., “Because the first pig built with straw, it fell down easily when the wolf blew on it”)					
38. Use stories to teach lessons or social rules (e.g., “This story teaches us that working hard and doing things carefully is important”)					

### Appendix A.4. Part 3: Knowing More About Your Child

The Adaptive Social Behavior Inventory (ASBI; Hogan et al., 1992) items are not reproduced here due to copyright. The ASBI is a 30-item parent-report measure with three subscales: Express (13 items; social expressiveness), Comply (10 items; cooperation), and Disrupt (7 items; disruptive behavior), rated on a 3-point scale (1 = rarely, 2 = sometimes, 3 = almost always). Please see Methods Section 2.2.5 for a complete description of the measure and scoring, and refer to the original publication (Hogan et al., 1992) for the full item list.

### Appendix A.5. Part 4: Parent Information

69. Your age range: □ 29 or younger □ 30–34 □ 35–39 □ 40–44 □ 45 or older
70. Are you a Singaporean citizen? □ Yes □ No (please specify): ___________
71. Highest level of education achieved:

**Education Level**	**Mother**	**Father**
No formal qualification	□	□
Primary school (PSLE or below)	□	□
Lower Secondary (Secondary 3 and below)	□	□
Secondary (Completed N or O levels)	□	□
Post Secondary (NITEC/A Levels/IB)	□	□
Diploma & Professional Certificates	□	□
University (Bachelor’s Degree)	□	□
Postgraduate (Master’s Degree)	□	□
Doctoral degree	□	□

72. Combined gross monthly family income (SGD):

□ Below $1000	□ $7000–$7999	□ $14,000–$14,999
□ $1000–$1999	□ $8000–$8999	□ $15,000–$19,999
□ $2000–$2999	□ $9000–$9999	□ $20,000–$24,999
□ $3000–$3999	□ $10,000–$10,999	□ $25,000–$29,999
□ $4000–$4999	□ $11,000–$11,999	□ $30,000 and above
□ $5000–$5999	□ $12,000–$12,999	
□ $6000–$6999	□ $13,000–$13,999	
